# Clinical characteristics and risk factors of organ failure and death in necrotizing pancreatitis

**DOI:** 10.1186/s12876-023-02651-4

**Published:** 2023-01-19

**Authors:** Liqing Yu, Fengwen Xie, Lingyu Luo, Yupeng Lei, Xin Huang, Xiaoyu Yang, Yong Zhu, Cong He, Nianshuang Li, Wenhua He, Yin Zhu, Nonghua Lu, Bingjun Yu

**Affiliations:** 1 Department of Gastroenterology, The First Affiliated Hospital of Nanchang University, 17 Yong Waizheng Street, Donghu District, Nanchang, 330006 Jiangxi Province China; 2 Second Clinical Medical College of Nanchang University, Nanchang, 330006 Jiangxi Province China

**Keywords:** Necrotizing pancreatitis, Risk factors, Clinical characteristics, Organ failure, Mortality

## Abstract

**Background:**

Organ failure (OF) and death are considered the most significant adverse outcomes in necrotizing pancreatitis (NP). However, there are few NP-related studies describing the clinical traits of OF and aggravated outcomes.

**Purpose:**

An improved insight into the details of OF and death will be helpful to the management of NP. Thus, in our research, we addressed the risk factors of OF and death in NP patients.

**Methods:**

We performed a study of 432 NP patients from May 2017 to December 2021. All patients with NP were followed up for 36 months. The primary end-points were risk factors of OF and death in NP patients. The risk factors were evaluated by logistic regression analysis.

**Results:**

NP patients with OF or death patients were generally older, had a higher APACHE II score, longer hospital stay, longer ICU stay, as well as a higher incidence of severe acute pancreatitis (SAP), shock and pancreatic necrosis. Independent risk factors related to OF included BMI, APACHE II score and SAP (*P* < 0.05). Age, shock and APACHE II score (*P* < 0.05) were the most significant factors correlated with the risk of death in NP patients. Notably, increased mortality was linked to the number of failed organs.

**Conclusions:**

NP is a potentially fatal disease with a long hospital or ICU stay. Our study indicated that the incidence of OF and death in NP patients was 69.9% and 10.2%, respectively. BMI, SAP, APACHE II score, age and shock are potential risk factors of OF and death in NP patients. Clinicians should focus on these factors for early diagnosis and appropriate therapy.

**Supplementary Information:**

The online version contains supplementary material available at 10.1186/s12876-023-02651-4.

## Introduction

Acute pancreatitis (AP) remains a common gastrointestinal disease, and the number of hospitalizations due to AP has been on the rise over the past decade [1]. According to the 2012 revised Atlanta classification criteria, AP can be classified as mild, moderate and severe [2]. Although most cases of pancreatitis are mild, around 20% will progress to severe pancreatitis with a mortality rate of 25%, characterized by persistent OF beyond 48 h and local complications including peripancreatic or pancreatic necrosis [2, 3]. Cases with necrosis exceeding 30% are classified as necrotizing pancreatitis, which accounts for 5–10% of acute pancreatitis cases [2].

Patients with sterile necrosis generally require symptomatic treatment, and the mortality is approximately 15% [4, 5]. By contrast, patients with infected necrosis usually have significantly increased mortality due to sepsis and multiple organ failure (MOP) [6]. Infected necrotizing pancreatitis (IPN) with surgical intervention has a mortality rate as high as 30%, while without any intervention, the mortality is close to 100% [7, 8]. Although the standard regimen for acute necrotizing pancreatitis (ANP) has gradually changed from early conventional surgery to endoscopic surgery and minimally invasive surgery (non-surgical treatment) over the past 10 years, with the continuous focus on being less invasive, patients with ANP still require considerable healthcare resources and have high mortality [9–15]. Hence, extensive further research is necessary to determine the clinical traits of this large population affected by necrotizing pancreatitis (NP) and to recognize the risk factors associated with worse outcomes.

Organ failure (OF), is thought to be a significant risk in AP which plays a key role in mortality [16–18]. A meta-analysis of 1478 patients with AP in 2010 revealed a total of 600 patients who developed OF with mortality as high as 30%, which is close to 10 times higher than in patients without OF [8, 19]. Therefore, an in-depth understanding of OF is crucial for the management of AP patients. Studies showed that specific characteristics of OF affect the clinical course and outcome. In clinical practice, renal, cardiovascular and respiratory failure are most commonly considered in AP [20]. Among these individual types of OF, respiratory failure is the most frequent, and cardiovascular failure leads to the worst outcomes, while the prognosis is significantly worse in case of multi-organ failure [10, 21, 22]. More importantly, the combination of OF and IPN doubles the risk of mortality, which suggests a close link between OF and necrosis [8, 23, 24]. This conjecture has been demonstrated in previous studies as both types of injury depend on the inflammatory response [25]. A prospective study of 104 patients with pancreatic necrosis indicated that the extent of necrosis and infected pancreatic necrosis were the two most significant factors associated with the development of OF [25]. The same conclusion was drawn by Isenmann et al. [26], but other studies reported discrepant observations [27, 28]. Accordingly, the effect of necrosis on OF remains uncertain, and there are also few comprehensive studies on other risk factors for OF in NP.

Taken together, improved insight into the details of OF and mortality will be helpful to the management of NP. Thus, in our research, we addressed the risk factors of OF and death in NP patients.

## Materials and methods

### Patients and assessments

**Fig. 1:**
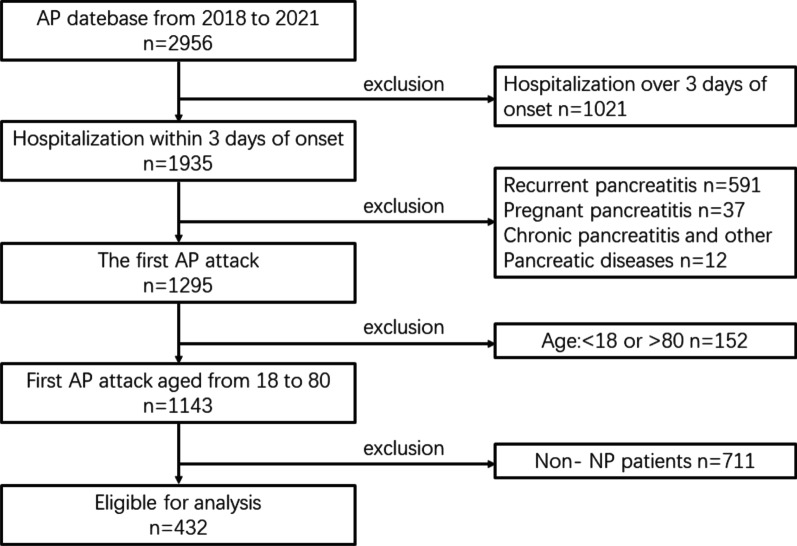
Study flowchart. *AP* acute pancreatitis, *NP* necrotizing pancreatitis

This was a retrospective study that was conducted in accordance with the Helsinki Declaration at the First Affiliated Hospital of Nanchang University in patients with NP from 2017 to 2021. All data were obtained from the AP database, with the approval of the ethics committee (Approval Number: 2011001). In total, 2,956 AP patients were screened, and 432 were included in this study. The exclusion criteria are depicted in Fig. 1. Informed consent linked to data storage or publication was obtained from patients in the database during hospitalization. Data included demographic characteristics (sex, age, body mass index [BMI]), etiology of AP, history of diabetes and hypertension, smoking, drinking, type of organ failure (i.e., cardiovascular, respiratory and renal failure), shock, operative treatment, total hospital stay and ICU stay, local complications (i.e., walled-off necrosis [WON], acute necrotic collection [ANC], pancreatic pseudocyst [PP], acute peripancreatic fluid collection [APFC]), death, laboratory results. The Acute Physiology and Chronic Health Evaluation II (APACHE II) score and severity of AP were calculated based on patient data.

### Diagnosis, definitions and treatments

Two or more of the following three characteristics were used as a basis to diagnose acute pancreatitis: (1) Acute onset of persistent epigastric pain, usually radiating backwards; (2) Amylase activity (or serum lipase activity) at least 3 times higher than the upper normal level; and (3) Typical signs of pancreatitis on abdominal imaging [2]. The etiology and severity of AP were determined according to the 2012 revised Atlanta classification criteria [2].

Local complications including WON, ANC, PP and APFC were diagnosed by contrast-enhanced computed tomography (CECT) [2, 29]. NP is diagnosed on contrast-enhanced cross-sectional imaging as the absence of pancreatic or peripancreatic enhancement. By contrast, normal pancreatic parenchyma demonstrated homogeneous enhancement with contrast administration. Patterns of necrosis in necrotizing pancreatitis may involve a combination of pancreatic and peripancreatic parenchyma, isolated peripancreatic necrosis, or, less commonly, isolated pancreatic necrosis. Local complications in NP include acute necrotic collections (ANCs) and walled-off necrosis (WON). An ANC is a pancreatic or peripancreatic collection with liquid and/or solid necrosis within the first 4 weeks from symptom onset. After 4 weeks, the pancreatic and peripancreatic necrosis matures to WON, after a well-defined wall of inflammatory reactive tissue has developed [30, 31]. OF was determined according to the modified Marshall scoring system, and the distinction between persistent and transient OF has been described before [2]. Death was recorded either as death during hospitalization, or in patients who were automatically discharged due to critical condition. Specific definitions are listed in Additional file 1: Table 1. In addition, we also recorded patient characteristics (age, sex, BMI), previous history (hypertension, diabetes, smoking, drinking), shock, operative treatment and hospitalization to assess risk factors in each group.

A key element in the early treatment of AP is fluid resuscitation. Continuous regional arterial infusion (CRAI) has been used to infuse fluids, protease inhibitors, and/or antibiotics, theoretically resulting in increased drug and fluid concentrations in the pancreatic tissue [31]. Aggressive hydration, defined as 250–500 ml per hour of isotonic crystalloid solution should be provided to all patients, unless cardiovascular and/or renal comorbidities exist. Early aggressive intravenous hydration is most beneficial in the first 12–24 h [6]. Lactated Ringer’s solution was the preferred isotonic crystalloid replacement fluid administered to the patients [6].

### Statistical analysis

Statistical analyses were performed in SPSS 24.0 (IBM Corp., Armonk, NY, USA). Measurement data with a normal distribution were expressed as means ± SD and analyzed using Student’s *t*-test or ANOVA (analysis of variance). Continuous variables were presented as the means ± standard deviations and compared using 1-way analysis of variance for normally distributed data. For skewed distributions, the data were presented as the median (interquartile range) and compared using the nonparametric Mann–Whitney U test. Categorical variables were described as percentages and compared using the Chi-squared test or Fisher’s exact test. Subsequently, logistic regression was performed to explore the risk factors correlated with organ failure and death. The association between the factors and dependent variables was first estimated through univariable logistic regression. Then, the pattern of related variables influencing outcomes was demonstrated using the Univariate odds ratios with 95% confidence intervals. Multivariate logistic regression analyses were performed to assess the potential determinants for severity stratification and prognostic prediction of AP by unadjusted and adjusted models, successively. Factors with *P* < 0.05 were then included in a multivariate logistic regression analysis. Statistical significance was defined as *P* < 0.05 (2-tailed).

## Results

### Comparison of patients with and without organ failure in necrotizing pancreatitis

From May 2017 to April 2021, 2956 patients were admitted to the First Affiliated Hospital of Nanchang University with the diagnosis of AP, and 432 met our inclusion criteria (Fig. 1). The patients were divided into two groups: 302 patients in the OF group and 130 patients in the OF-free group. A total of 277 males accounted for 64.1% of the study population, while 155 females accounted for 35.9%. The average age was 48.1 ± 14.2 years for all NP patients. Compared to patients without OF, the OF patients were older (49.3 ± 14.4 vs. 45.5 ± 13.5, *P* < 0.05), had a higher BMI (25.3 ± 3.7 vs. 24.1 ± 2.9, *P* < 0.001), higher APACHE II score (10.9 ± 4.6 vs. 8.5 ± 3.2, *P* < 0.001), longer total hospital stay (24.9 ± 23.4 vs. 9.5 ± 7.8, *P* < 0.001) and longer ICU stay (11.0 ± 16.6 vs. 0.5 ± 1.8, *P* < 0.001) (Table 1). In past medical history, incidence of diabetes in the OF patients was higher than in the patients without OF (*P* < 0.05). Notably, both death and shock occurred only in the OF group. A significant difference was found in the severity of AP among the two groups (*P* < 0.001). Among all NP patients and OF patients, SAP patients accounted for a maximum of 47.7% and 67.2% respectively, while in the OF-free group the MSAP patients made up the majority (90.0%).

**Table 1:** Characteristics of acute necrotizing pancreatitis patients with or without organ failure

	All patients (n = 432)	OF = 302 (69.9%)	OF-free = 130 (30.1%)	*P* values
Age (years)	48.1 ± 14.2	49.3 ± 14.4	45.5 ± 13.5	0.011*
*Sex*				0.888^‡^
Male	277 (64.1%)	193 (63.9%)	84 (64.6%)	
Female	155 (35.9%)	109 (36.1%)	46 (35.4%)	
BMI	24.9 ± 3.5	25.3 ± 3.7	24.1 ± 2.9	0.000*
*Severity of AP*				0.000†
MAP	15 (3.5%)	5 (1.7%)	10 (7.7%)	
MSAP	211 (48.8%)	94 (31.1%)	117 (90.0%)	
SAP	206 (47.7%)	203 (67.2%)	3 (2.3%)	
*Etiology of AP*				0.168^‡^
Biliary	155 (35.9%)	114 (37.7%)	41 (31.5%)	
HTG	164 (38.0%)	109 (36.1%)	55 (42.3%)	
Alcohol	28 (6.5%)	17 (5.6%)	11 (8.5%)	
Comprehensive	36 (8.3%)	30 (9.9%)	6 (4.6%)	
Other	47 (10.9%)	30 (9.9%)	17 (13.1%)	
Pre-existent Hypertension	78 (18.1%)	61 (20.2%)	17 (13.1%)	0.078^‡^
Pre-existent diabetes	46 (10.6%)	38 (12.6%)	8 (6.2%)	0.047^‡^
Smoking	134 (31.0%)	102 (33.8%)	32 (24.6%)	0.059^‡^
Drinking	156 (36.1%)	115 (38.1%)	41 (31.5%)	0.194^‡^
shock	49 (11.3%)	49 (16.2%)	0	0.000^‡^
*Operative treatment*				
PCD	127 (29.4%)	121 (40.1%)	6 (4.6%)	0.000^‡^
ERCP	30 (6.9%)	18 (6.0%)	12 (9.2%)	0.220^‡^
APACHE II	10.2 ± 4.3	10.9 ± 4.6	8.5 ± 3.2	0.000*
ICU admission	222 (51.4%)	209 (69.2%)	13 (10.0%)	0.000^‡^
ICU stay, days	7.9 ± 14.7	11.0 ± 16.6	0.5 ± 1.8	0.000*
LOS, days	20.2 ± 21.2	24.9 ± 23.4	9.5 ± 7.8	0.000*
Death	44 (10.2%)	44 (14.6%)	0	0.000†

Continuous variables are presented as the mean (standard deviation). Categorical variables are presented as number (percentage)

Acute Physiology and Chronic Health Evaluation II = APACHE II. APACHE II scores range from 2 to 17, and higher scores refer to more severe disease. Organ failure = OF. Acute pancreatitis = AP. Body mass index = BMI. Mild acute pancreatitis = MAP. Moderately severe acute pancreatitis = MSAP. Severe acute pancreatitis = SAP. Hypertriglyceridemia = HTG. Percutaneous drainage = PCD. Endoscopic retrograde cholangiopancreatography = ERCP. Intensive care unit = ICU. Length of stay = LOS

*Student’s *t* test

^‡^Pearson Chi-Square test

^†^Fisher's exact test

In terms of etiology, hypertriglyceridemia was the main cause in all included NP patients (38.0%), and similar odds of hypertriglyceridemia as the etiology of NP were found in the OF-free group (42.3%). In addition to hypertriglyceridemia, a major cause in the OF group was biliary complications (37.7%). As shown in Fig. 1, there were no significant differences in the etiology of AP among the two groups. ERCP is considered one of the least common causes of AP [32], and the difference between the two groups was not statistically significant (*P* < 0.05). As one of the prime examples of surgical modalities ranging from open surgery to minimally invasive surgery, a total of 127 patients underwent a PCD [31]. Among them, 121(40.1%) were in the OF group and 6(4.6%) in the OF-free group. Moreover, the difference was highly statistically significant (*P* < 0.001).

In our study, the incidence of ANC in the OF group was 72.2%, and the ratio in OF-free group was 92.3%. ANC infection was detected in 67(22.2%) patients in the OF group compared to 4 (3.1%) patients in the OF-free group. WON and WON infection seldom occurred in either group. There were statistically significant differences between the two groups in local complication characteristics (*P* < 0.001). More information is provided in Additional file 1: Table 2.

### Comparison of death and surviving patients

To explore the impact of various factors on the mortality of NP patients, we divided all patients into two groups according to their survival status (Table 2). The death group included 44(10.2%) patients, and the surviving group included 388(89.8%) patients. The mean age was 48.1 ± 14.2 years, and there was a highly significant difference in age between the two groups (56.8 ± 13.8 years vs. 47.1 ± 14.0 years, *P* < 0.001), while sex, BMI, etiology and past medical history (i.e., hypertension, diabetes, smoking and drinking) were not significantly different (*P* > 0.05). Further analysis indicated that the death patients had higher APACHE II scores (13.0 ± 4.8 vs. 9.9 ± 4.2, *P* < 0.001), longer total hospital stay (26.7 ± 20.7 vs. 19.5 ± 21.2, *P* < 0.05) and longer ICU stay (20.7 ± 19.4 vs. 6.4 ± 13.4, *P* < 0.001). In addition, we found that NP patients were more prone to OF (69.9%), but the probability of MOF (19.7%) and shock (11.3%) was relatively low. Patients who experienced shock (27 vs. 22, *P* < 0.001) were more likely to die. The incidence of single or multiple OF (i.e., respiratory failure, renal failure and circulatory failure) was significantly higher in the death group than in the surviving group (*P* < 0.001) (Additional file 1: Tables 2 and 3). In terms of operative treatment, PCD was more accepted among patients, and there were statistically significant differences in PCD between the two groups (4.5% vs. 7.2%, *P* < 0.001). The main local complication among the NP patients was ANC (78.2%), with an incidence in the death group of 40.9%, and an incidence in surviving group of 82.5%. ANC infection was found in 20(45.5%) patients in the death group, compared to 51 (13.1%) patients in the surviving group. WON and WON infection were rare in both groups. Highly statistically significant differences between the two groups were found in local complications (*P* < 0.001) (Additional file 1: Table 3).

**Table 2:** Characteristics of acute necrotizing pancreatitis patients died or not

	All patients (n = 432)	Death = 44 (10.2%)	Surviving = 388 (89.8%)	*P* values
Age (years)	48.1 ± 14.2	56.8 ± 13.8	47.1 ± 14.0	0.000*
*Sex*				0.687^‡^
Male	277 (64.1%)	27 (61.4%)	250 (64.4%)	
Female	155 (35.9%)	17 (38.6%)	138 (35.6%)	
BMI	24.9 ± 3.5	24.9 ± 3.5	24.9 ± 3.5	0.949*
*Severity of AP*				0.000^†^
MAP	15 (3.5%)	1 (2.3%)	14 (3.6%)	
MSAP	211 (48.8%)	3 (6.8%)	208 (53.6%)	
SAP	206 (47.7%)	40 (90.9%)	166 (42.8%)	
*Etiology of AP*				0.268^†^
Biliary	155 (35.9%)	22 (50.0%)	133 (34.3%)	
HTG	164 (38.0%)	11 (25.0%)	153 (39.4%)	
Alcohol	28 (6.5%)	4 (9.1%)	24 (6.2%)	
Comprehensive	36 (8.3%)	4 (9.1%)	32 (8.2%)	
Other	47 (10.9%)	3 (6.8%)	44 (11.3%)	
Pre-existent Hypertension	78 (18.1%)	10 (22.7%)	68 (17.5%)	0.395^‡^
Pre-existent Diabetes	46 (10.6%)	5 (11.4%)	41 (10.6%)	0.871^‡^
Smoking	134 (31.0%)	12 (27.3%)	122 (31.4%)	0.571^‡^
Drinking	156 (36.1%)	16 (36.4%)	140 (36.1%)	0.971^‡^
Organ failure	302 (69.9%)	44 (100.0%)	258 (66.5%)	0.000^†^
Multiple organ failure(MOF)	85 (19.7%)	35 (79.5%)	50 (12.9%)	0.000^‡^
Shock	49 (11.3%)	27 (61.4%)	22 (5.7%)	0.000^‡^
*Operative treatment*				
PCD	127 (6.9%)	32 (4.5%)	95 (7.2%)	0.000^‡^
ERCP	30 (29.4%)	2 (72.7%)	28 (24.5%)	0.728^†^
APACHE II	10.2 ± 4.3	13.0 ± 4.8	9.9 ± 4.2	0.000*
ICU admission	222 (51.4%)	41 (93.2%)	181 (46.6%)	0.000^†^
ICU stay, days	7.9 ± 14.7	20.7 ± 19.4	6.4 ± 13.4	0.000*
LOS, days	20.2 ± 21.2	26.7 ± 20.7	19.5 ± 21.2	0.034*

Continuous variables are presented as the mean (standard deviation). Categorical variables are presented as number (percentage)

Acute Physiology and Chronic Health Evaluation II = APACHE II. APACHE II scores range from 2 to 17, and higher scores refer to more severe disease. Acute pancreatitis = AP. Body mass index = BMI. Mild acute pancreatitis = MAP. Moderately severe acute pancreatitis = MSAP. Severe acute pancreatitis = SAP. Hypertriglyceridemia = HTG. Percutaneous drainage = PCD. Endoscopic retrograde cholangiopancreatography = ERCP. intensive care unit = ICU. Length of stay = LOS. Multiple organ failure = MOF

*Student’s *t* test

^‡^Pearson Chi-Square test

^†^Fisher's exact test

### Univariate and multivariate regression analyses of risk factors for clinical outcomes

Logistic regression analysis was used to investigate the risk factors for OF. The univariate analysis indicated that older age (OR 0.98, 95% CI 0.97–1.00, *P* = 0.012), higher BMI (OR 0.90, 95% CI 0.84–0.96, *P* = 0.001), higher APACHE II score (OR 0.86, 95% CI 0.82–0.91, *P* = 0.012), SAP (OR 0.01, 95% CI 0.00–0.04, *P* < 0.001) and experience of PCD (OR 0.07, 95% CI 0.03–0.17, *P* < 0.001) were risk factors for OF (*P* < 0.05, Table 3). In the multivariate analysis, higher BMI (OR 0.87, 95% CI 0.79–0.95, *P* = 0.003), higher APACHE II scores (OR 0.91, 95% CI 0.83–0.99, *P* = 0.022) and SAP (OR 0.01, 95% CI 0.00–0.05, *P* < 0.001) were revealed as independent risk factors for OF (*P* < 0.05, Table 3). Notably, there was no clear link between local complications and OF (*P* < 0.05, Additional file 1: Table 4).

**Table 3:** Univariate and multivariate analyses of risk factors for organ failure in Acute necrotizing pancreatitis

Variable	Univariate analyses		Multivariate analyses	
	OR (95% CI)	*P*	OR (95% CI)	*P*
Age (years)	0.98 (0.97–1.00)	0.012	0.98 (0.96–1.00)	0.053
Sex (male/female)	1.03 (0.67–1.58)	0.888		
BMI	0.90 (0.84–0.96)	0.001	0.87 (0.79–0.95)	0.003
*Severity of AP*				
SAP	0.01 (0.00–0.04)	0.000	0.01 (0.00–0.05)	0.000
MSAP	0.62 (0.21–1.89)	0.401	0.52 (0.15–1.76)	0.290
MAP	Ref (1.00)		Ref (1.00)	
*Etiology of AP*				
Biliary	581,150,717.80 (0.00–)	0.999		
HTG	815,353,546.90 (0.00–)	0.999		
Alcohol	1,045,571,019.00 (0.00–)	0.999		
Comprehensive	323,176,496.80 (0.00–)	0.999		
Other	915,666,740.80 (0.00–)	0.999		
No	Ref (1.00)			
Pre-existent Hypertension	0.59 (0.33–1.06)	0.080		
Pre-existent Diabetes	0.46 (0.21–1.01)	0.052		
Smoking	0.64 (0.40–1.02)	0.060		
Drinking	0.75 (0.48–1.16)	0.195		
shock	0.00 (0.00-)	0.997		
*Operative treatment*				
PCD	0.07 (0.03–0.17)	0.000	0.46 (0.16–1.34)	0.154
ERCP	1.61 (0.75–3.44)	0.224		
APACHE II	0.86 (0.82–0.91)	0.000	0.91 (0.83–0.99)	0.022

odds ratio = OR. 95% confidence interval = 95% CI. Acute Physiology and Chronic Health Evaluation II = APACHE II. APACHE II scores range from 2 to 17, and higher scores refer to more severe disease. Acute pancreatitis = AP. Body mass index = BMI. Mild acute pancreatitis = MAP. Moderately severe acute pancreatitis = MSAP. Severe acute pancreatitis = SAP. Hypertriglyceridemia = HTG. Percutaneous drainage = PCD. Endoscopic retrograde cholangiopancreatography = ERCP

Subsequently, univariate analysis was conducted to evaluate the risk factors for death. Older age (OR 0.95, 95% CI 0.93–0.98, *P* < 0.001), MOF (OR 0.04, 95% CI 0.02–0.08, *P* < 0.001), shock (OR 0.04, 95% CI 0.02–0.08, *P* < 0.001), performance of PCD (OR 0.12, 95% CI 0.06–0.25, *P* < 0.001) and higher APACHE II scores (OR 0.85, 95% CI 0.80–0.92, *P* < 0.001) were significantly linked to a higher mortality in the univariate analysis (*P* < 0.05, Table 4). Among the three types of OF, only renal failure (OR 0.05, 95% CI 0.02–0.10, *P* < 0.001) and circulatory failure (OR 0.03, 95% CI 0.01–0.17, *P* < 0.001) were significantly associated with death (*P* < 0.05, Additional file 1: Table 5).

**Table 4:** Univariate and multivariate analyses of risk factors for death in Acute necrotizing pancreatitis

Variable	Univariate analyses		Multivariate analyses	
	OR (95% CI)	*P*	OR (95% CI)	*P*
Age (years)	0.95 (0.93–0.98)	0.000	0.93 (0.90–0.96)	0.000
Sex (male/female)	1.14 (0.60–2.17)	0.688		
BMI	1.00 (0.92–1.10)	0.949		
*Severity of AP*				
SAP	0.30 (0.04–2.32)	0.247		
MSAP	4.95 (0.48–50.75)	0.178		
MAP	Ref (1.00)			
*Etiology of AP*				
Biliary	0.00 (0.00–)	0.999		
HTG	0.00 (0.00–)	0.999		
Alcohol	0.00 (0.00–)	0.999		
Comprehensive	0.00 (0.00–)	0.999		
Other	0.00 (0.00–)	0.999		
No	Ref (1.00)			
Pre-existent Hypertension	0.72 (0.34–1.53)	0.397		
Pre-existent Diabetes	0.92 (0.34–2.47)	0.871		
Smoking	1.22 (0.61–2.46)	0.571		
Drinking	0.99 (0.52–1.89)	0.971		
Organ failure	0.00 (0.00–)	0.996		
Multiple organ failure (MOF)	0.04 (0.02–0.08)	0.000	0.15 (0.00–6.23)	0.319
shock	0.04 (0.02–0.08)	0.000	0.13 (0.05–0.34)	0.000
*Operative treatment*				
PCD	0.12 (0.06–0.25)	0.000	0.39 (0.14–1.06)	0.064
ERCP	1.63 (0.38–7.10)	0.513		
APACHE II	0.85 (0.80–0.92)	0.000	1.11 (1.00–1.23)	0.050

odds ratio = OR. 95% confidence interval = 95% CI. Acute Physiology and Chronic Health Evaluation II = APACHE II. APACHE II scores range from 2 to 17, and higher scores refer to more severe disease. Acute pancreatitis = AP. Body mass index = BMI. Mild acute pancreatitis = MAP. Moderately severe acute pancreatitis = MSAP. Severe acute pancreatitis = SAP. Hypertriglyceridemia = HTG. Percutaneous drainage = PCD. Endoscopic retrograde cholangiopancreatography = ERCP. Multiple organ failure = MOF

To avoid confounding factors, multivariate logistic regression analyses of risk factors for death were performed. In the multivariate analysis, death was associated with older age (OR 0.93, 95% CI 0.90–0.96, *P* < 0.001), shock (OR 0.13, 95% CI 0.05–0.34, *P* < 0.001) and APACHE II (OR 1.11, 95% CI 1.00–1.23, *P* < 0.001) (*P* < 0.05, Table 4).

### Type of organ failure and impact on mortality

The mortality of patient groups stratified by OF and combinations is shown in Table 5. Among the 302 patients with OF, 209 patients were diagnosed with persistent OF with 19.6% mortality. Transient OF occurred in only 93 patients and was linked to 9.7% mortality. Failure of the respiratory system was the most common in both the transient and persistent OF groups, with mortality rates of 2.4% and 5.5%, respectively, followed by failure of the renal system without records of death. Interestingly, there was no combination of cardiovascular failure with other types of OF in the transient OF group, and the only observed OF combination (i.e., combined respiratory and renal failure) was associated with a mortality of 20.0%. The group with persistent OF had the same most common combination type with greater mortality rates of 38.9%. Notably, increased mortality was linked to the number of failed organs: 9/217(4.1%) for one, 30/79(38.0%) for two and 5/6(83.3%) for three. In total, 6 patients had simultaneous failure of all three systems, which was associated with the highest mortality rate of 83.3%.

**Table 5:** Mortality in different subgroups in 302 patients with organ failure

Subgroups	Mortality (%)		
	Transient organ failure (3/93, 9.7%)	Persistent organ failure (41/209, 19.6%)	Total (44/302, 14.6%)
*Single organ failure*			
Any organ system	2/88 (2.3%)	7/129 (5.4%)	9/217 (4.1%)
Cardiovascular	–	–	–
Respiratory	2/85 (2.4%)	7/128 (5.5%)	9/213 (4.2%)
Renal	0/3	0/1	0/4
*Multiple organ failure (any two or more organ systems)*	1/5 (20.0%)	34/80 (42.5%)	35/85 (41.2%)
Any two organ systems	1/5 (20.0%)	29/74 (39.2%)	30/79 (38.0%)
Cardiovascular and respiratory	–	1/2 (50.0)	1/2 (50.0)
Respiratory and renal	1/5 (20.0%)	28/72 (38.9%)	29/77 (37.7%)
Renal and cardiovascular	–	–	–
All three organ systems	–	5/6 (83.3%)	5/6 (83.3%)

The data represent the longest persistent episode of organ failure in each system. If the duration of the episodes of different organ systems is equal between each other, the organ systems involved in the first episode was selected

## Discussion

NP is a potentially life-threatening disease, with an especially poor prognosis in cases of infection and OF secondary to peripancreatic necrosis [8, 23, 24]. According to the determinant-based classification (DBC) of AP, critical acute pancreatitis (CAP), infected pancreatic necrosis (IPN) and persistent organ failure (POF) beyond 48 h are hallmark events of the newly-defined category [33]. Prospective observational studies from different centers reported that CAP may occur in only 2.2–6.6% of AP, while being associated with significant mortality ranging from 17.7 to as high as 87.5% [34]. Although management strategies have changed from early surgery to conservative treatment including internal medicine, endoscopic surgery and minimally invasive surgery, which achieved a better prognosis in NP, a high incidence still remains [11, 13]. For these reasons, increasing knowledge on risk factors for worse outcome of NP is important for clinical practice.

To our knowledge, few studies conducted a detailed analysis of the effects of different factors on OF and outcomes in NP to date. Within this context, data from 432 patients with NP were retrospectively collected from the AP database at the First Affiliated Hospital of Nanchang University. We investigated the clinical outcomes, and assessed the risk factors associated with high mortality and OF in NP patients.

As described in the results section, the present study found that older age was a significant risk factor for death in NP. This is in line with a previous study that confirmed the connection between increasing age and higher AP-related mortality in detail [35]. A possible explanation would be the decreasing resistance to various physiological stresses such as inflammation, infection, and oxidative damage in the elderly [36]. In addition, elderly NP patients are deficient in protective pancreatitis-associated proteins and have a higher production of pro-inflammatory factors in case of systemic inflammatory response syndrome (SIRS) and sepsis compared to younger patients [37–39]. According to the World Health Organization (WHO), over 13% of the global adult population were considered “obese” (BMI ≥ 30) in 2016, and the growing global obesity rate is alarming due to numerous comorbidities such as hyperlipidemia or osteoarthritis, which are associated with increasing medical expenses [40]. Several previous studies reported that a high BMI was directly linked to increased risk of MOF, local complications and poor prognosis in AP [41–44]. In our multivariate logistic analysis, higher BMI was independently associated with OF (OR 0.87, 95% CI 0.79–0.95, *P* = 0.003) which is probably related to the unsaturated fatty acids (UFAs) generated by the lipolysis of visceral fat [45]. However, no significant association was found between higher BMI and death. This was different from a recent study indicating that morbid obesity (BMI > 30 kg/m^2^) is associated with an increased risk of in-hospital mortality (OR 2.58, 95% CI 1.21–5.53, *P* = 0.02). We speculated that this discrepancy may be attributable to the different populations used to calculate the mortality, since the former included automatically discharged patients into the death group, while the latter only calculated in-hospital mortality. However, these results for NP were based on only 2 studies, and further studies are necessary for firm conclusions.

As the primary tool used to estimate the severity of pancreatitis, the Acute Physiology and Chronic Health Evaluation II (APACHE II) score consists of age index, acute physiology and chronic health evaluation [46]. A previous study revealed that the APACHE-II score was correlated with the prognosis of SAP patients [47]. In another retrospective study, Kazuhiro Minami et al. compared the clinical features between death and surviving patients with a diagnosis of NP, and determined the role of higher APACHE-II score as independent risk factor of death [36]. Similarly, we also found that the APACHE-II score is an independent risk factor for mortality and OF (OR 1.11, 95% CI 1.00–1.23, *P* = 0.050) (OR 0.91, 95% CI 0.83–0.99, *P* = 0.022). In addition, SAP was a robust risk factor for OF in our study, which is in agreement with the fact that OF is one of the crucial characteristics of SAP [2].

In terms of etiology, biliary-induced pancreatitis is considered to be the most significant etiological factor of AP in China, especially in the elderly who have a much larger diameter of the bile duct and higher incidence of gallstones [29, 38, 48–50]. Despite such a wide base of biliary causes, only one-sixth of the elderly in our NP cohort made the proportion of cases with a biliary etiology rank second, while the most common cause in our study was hyperlipidemia. Several observations in AP patients indicated that hypertriglyceridemia-induced pancreatitis had generally a more severe clinical course, including an increased incidence of OF and pancreatic necrosis [51–53]. A retrospective cohort study even suggested that elevated serum TGs in AP patients were independently correlated with OF [54]. The more severe outcomes observed in hyperlipidemia are probably caused by the lipolysis of serum triglycerides, which leads to an excess of free fatty acids (FFAs), resulting in acinar injury, endothelial dysfunction and activation of the inflammatory cascade [55–57]. It therefore stands to reason that the highest proportion of NP etiology in our dataset was HTG (38.0%), as HTG is certainly associated with profound damage. However, although our analysis also noted a higher rate of OF and higher mortality regardless of HTG status, there was no significant difference in terms of NP clinical course between different etiologies according to our data. This finding was consistent with a recent large, single-institution study of 676 patients with NP [58]. One potential explanation for this may be that once a patient with AP develops NP, worst clinical outcomes are mainly explained by other risk factors (such as hemorrhage [59], colon involvement [60], abdominal free fluid [61], low skeletal muscle density [62], etc.) rather than etiology. For this reason, we still do believe that hypertriglyceridemia is an aggravating risk factor for death and OF in AP as well as NP patients, which should be confirmed in the future by more detailed studies.

It used to be generally believed that high mortality was mainly related to concomitant surgical or medical diseases, rather than complications owing to the pathological process of AP [63]. However, our data did not recognize differences in preexisting chronic diseases between NP outcomes, nor did preexisting chronic diseases act as independent risk factors for OF or mortality. This conclusion was consistent with a previous study by Quero et al., who found no strict relation between the Charlson score (an index used to predict the risk of death resulting from comorbid disease) and mortality [64, 65].

Pancreatic necrosis is a common complication which may be categorized as acute necrotic collection (ANC) or walled-off necrosis (WON). The former usually develops within 4 weeks of the pancreatitis course, consisting of peripancreatic effusion and accumulation of pancreatic or peripancreatic necrotic material [66]. Nearly 50% of ANC cases proceed to develop walled-off necrosis (WON) [2, 67]. The collected pancreatic fluid and necrotic material are sterile in the early stages, but it becomes infected in approximately one-third of patients during the course of disease [68]. Our study also found a similar infection rate of 19.5%. Moreover, it seems that the incidence of IPN is generally correlated with the prevalence of OF and even death in acute pancreatitis [8, 26, 69]. In comparison with the sterile group in our study, there was also a higher proportion of infected necrosis both in the OF and death group, with significant differences. Notably, a German prospective cohort study from 1999 comprising 300 NP patients identified infected necrosis as the most significant risk factor for OF [26]. However, multivariate logistic regression analysis failed to confirm infected necrosis as an independent prognostic factor for OF in our study, although there was a statistically significant difference between the two groups. We suspect that the different conclusions between the two studies might mainly stem from changes of diagnostic criteria and treatment strategies over the past 20 years, as well as the uncontrollable nature of prospective studies. On the other hand, we also observed that infection was not independently associated with mortality. The results of a large prospective study support our findings after adjusting for OF [70].

Here, we mainly focused on OF as one of the most important complications of pancreatitis. Consistent with the original hypothesis, the occurrence of OF (i.e. respiratory failure, renal failure and circulatory failure) was associated with an increased risk of death, with respiratory failure ranking first among the proximal causes of mortality. The obviously higher rate of respiratory failure (69.0%) was the major cause. This is in line with a Scottish report, which found that pulmonary dysfunction was most prevalent in AP [21]. Another explanation is likely to involve AP-induced systemic inflammation. A large number of inflammatory factors reach the pulmonary tissue through systemic circulation, leading to impaired alveolar gas exchange, reduced ventilation and increased plasma protein permeability [71–73]. One consequence of the massive damage to the air-filled aerated lung tissue is the development of severe hypoxemia, which exacerbates systemic dysfunction and further increases the risk of death [74]. Broadly comparable to these earlier studies, MOF was identified as a determinant linked to high mortality in NP patients by Trikudanathan et al. [62]. In contrast with their findings, OF was not identified as an independent risk factor for mortality in our multivariate logistic analysis. However, the included population (only patients aged 18–80 years were selected) and the endpoint (default definition of patients who were automatically discharged from the hospital as dead) in our research may have resulted in selection bias and potential confounding. Thus, there is still reason to believe that OF is a risk factor for death. According to our analysis, increased mortality was related to the number of failed organs, with a ratio of 9/217(4.1%) for one, 30/79(38.0%) for two and 5/6(83.3%) for three. This observation was in agreement with a cohort study of patients with NP from 21 Dutch hospitals [10]. The Dutch study revealed an impact of OF on mortality in necrotizing pancreatitis, but there was no connection between mortality and the duration of OF. However, two other studies reported findings in contrast with the Dutch data [75, 76], as there was a significant relationship between mortality and the duration of OF in these two studies. A possible explanation for this divergence could be the study design, as the latter studies were mostly performed on retrospective data, while the Dutch team conducted a prospective study that may have correctly deduced a causal link between the duration of OF and death in contrast to retrospective studies. Another cause could be data collection itself, which is a long and tedious process including the recording of the start, end and type of OF on a daily basis. It is conceivable that errors in this complex task can easily lead to inconsistencies in the results of analysis. Unfortunately, we did not document the dynamics of OF in detail to examine these different results. Further multicenter prospective studies should be performed to provide more precise data to make up for these shortcomings.

There are several strengths in this study. Firstly, we focused on the worse prognosis related to NP. Broader insights were obtained provided into the clinical characteristics and risk factors of OF and death in necrotizing pancreatitis, and no similar studies have been published to date. We found that MOF, age and shock could potentially be used as simple parameters to identify high-risk mortality. BMI and SAP were identified independent factors of OF in patients with necrotizing pancreatitis, and NP patients with higher APACHE II scores had more severe outcomes. The identification of patient characteristics related to OF and mortality may help clinicians recognize individuals at greatest risk of worse prognosis during hospitalization. Although this was a retrospective study based on data derived from a single center, given that all NP patients over a 5-year duration were consecutively enrolled in our study, it is likely that our observation represents the majority of current clinical experience. However, we do acknowledge some limitations of our study. Firstly, a large number of severe cases were referred to our tertiary hospital, which may have resulted in potential confounding factors related to different initial treatment. Another limitation is that we did not documented the dynamics of OF in detail to solve disputes already raised in the existing literature, such as the impact of the duration of OF on mortality.

## Conclusions

Our results suggest that BMI, SAP and APACHE II score are independent risk factors for OF in patients with NP. Furthermore, MOF, age, shock and APACHE II score could potentially be used as a simple index to identify patients at high risk of mortality. However, sex, etiology and past history (pre-existing hypertension, pre-existing diabetes, smoking and drinking) were not associated with differences in mortality or OF. Clinicians should focus on these features to effectively aid in risk stratification of hospitalized patients and allocate intensive care resources accordingly.

## Supplementary Information

**Additional file 1: Supplementary Table 1.** Definitions of diagnostic criteria. **Supplementary Table 2.** Characteristics of Acute necrotizing pancreatitis patients with or without organ failure. **Supplementary Table 3.** Characteristics of Acute necrotizing pancreatitis patients died or not. **Supplementary Table 4.** Univariate and multivariate analyses of risk factors for organ failure in acute necrotizing pancreatitis.

## Data Availability

The datasets used and/or analyzed during the current study are available from the corresponding author on reasonable request.

## Abbreviations

AP: Acute pancreatitis
RAP: Recurrent acute pancreatitis
CP: Chronic pancreatitis
SAP: Severe acute pancreatitis
CECT: Contrast-enhanced computed tomography
ERCP: Endoscopic retrograde cholangiopancreatography
HTG: Hypertriglyceridemia
CT: Computed tomographic
APACHEII: Acute Physiology and Chronic Health Evaluation II
FFAs: Free fatty acids
