# Supplementary material for: Clinical characteristics and risk factors of organ failure and death in necrotizing pancreatitis
Source: BMC Gastroenterol. 2023 Jan 19;23:19. doi: 10.1186/s12876-023-02651-4 (PMC9850524; doi:10.1186/s12876-023-02651-4)
Supplement: Supplementary file 1 — Additional file 1: Supplementary Table 1. Definitions of diagnostic criteria. Supplementary Table 2. Characteristics of Acute necrotizing pancreatitis patients with or without organ failure. Supplementary Table 3. Characteristics of Acute necrotizing pancreatitis patients died or not. Supplementary Table 4. Univariate and multivariate analyses of risk factors for organ failure in acute necrotizing pancreatitis. [file 12876_2023_2651_MOESM1_ESM.docx]

**Supplementary Table 1. Definitions of diagnostic criteria**

| Diagnostic criteria | **Definition** |
| --- | --- |
| **AP** | Two or more of the following three characteristics are required to diagnose the acute pancreatitis: (1) acute onset of persistent epigastric pain, usually radiating backwards; (2) the amylase activity (or serum lipase activity) at least 3 times more than the upper normal level; and (3) typical signs of pancreatitis on abdominal imaging. |
| **MAP** | Absence of (local or systemic) complications and organ failure |
| **MSAP** | Transient organ failure with/or (local or systemic) complications |
| **SAP** | Persistent organ failure with/or (local or systemic) complications |
| **Etiology of AP** |  |
| Biliary pancreatitis | At least one of the following criteria is present: sludge and/or gallstones on CT or ultrasonography; dilated CBD on CT or ultrasonography (diameter > 10 mm in age > 75 years and diameter > 8 mm in age≤75years); two of the following three experiments abnormal: level of serum bilirubin concentration higher than 2.3 mg/dL, alanine amino transferase (ALT) activity beyond 100 U/L with an ALT activity higher than aspartate aminotransferase (AST) activity; γ-glutamyl-transferase (GGT) activity higher than 45 U/L with alkaline phosphatase activity higher than 195 U/L. |
| HTG-pancreatitis | Serum triglycerides beyond 1,000 mg/dl in the lack of a history of significant alcohol use and/or gallstones /or other known cause of AP |
| Alcoholic pancreatitis | Have a history of heavy drinking (> 50 grams per day) for more than 5 years. |
| Others | Pancreatitis of unknown etiology. |
| **Pancreatic complication (CECT criteria)** |  |
| ANC | Occurs only in case of acute necrotizing pancreatitis. Refer to an un-encapsulating collection intrapancreatic and/or extrapancreatic, containing variable quantity of both fluid and necrosis related to necrotizing pancreatitis |
| ANC infection | The occurrence of extraluminal gas in necrotic areas or positive culture of fine-needle aspirate. |
| WON | Usually occurs >4 weeks after the onset of necrotizing pancreatitis. With mature, encapsulated collection intrapancreatic and/or extrapancreatic, and heterogeneous of liquid or non-liquid density (some may reveal  homogeneous) |
| WON infection | Surrounded by radiologically identifiable capsules and the occurrence of extraluminal gas in necrotic areas or positive culture of fine-needle aspirate. |
| APFC | Occurs only in case of interstitial oedematous pancreatitis. Refer to an un-encapsulating collection with fluid density, limited by peripancreatic fascial planes |
| APFC infection | APFC in interstitial oedematous pancreatitis and the occurrence of extraluminal gas in necrotic areas or positive culture of fine-needle aspirate. |
| PPC | Usually occurs >4 weeks after the onset of interstitial oedematous pancreatitis. With encapsulated collection of only fluid density usually outside the pancreas. |
| **Necrotizing pancreatitis** | Absence of pancreas enhancement via intravenous contrast agent and/or observation of peripancreatic necrosis (ANC or WON). |
| **Organ failure** |  |
| Pulmonary failure | Need for mechanical ventilation or PaO2<60 mmHg although FIO2 of 0.30 |
| Circulatory failure | Need for inotropic catecholamine or circulatory systolic blood pressure <90 mmHg although enough fluid resuscitation |
| Renal failure | Level of creatinine >177 μmol/L after rehydration or new require for haemodialysis or haemofiltration |
| **Multiple organ failure** | Sequential failure occurs in two or more organs. |
| **Transient organ failure** | The same organ system failed for less than 48 hours. |
| **Persistent organ failure** | The same organ system failed for 48 hours or more. |
| **Shock** | A systolic blood pressure of 80 mm/Hg or less over a period of more than 15 minutes. |
| **Death** | Death owing to pancreatitis-related complications during hospitalization and the patients who are automatically discharged due to critical condition |

**Abbreviations:** Acute pancreatitis=AP. Mild acute pancreatitis=MAP. Moderately severe acute pancreatitis=MSAP. Severe acute pancreatitis=SAP. Contrast-enhanced computed tomography=CECT. Pancreatic pseudocyst=PPC. Walled-off necrosis=WON. Acute peripancreatic fluid collection=APFC. Acute necrosis collection=ANC. [Hypertriglyceridemia](javascript:;)=HTG

**Supplementary Table 2. Characteristics of Acute necrotizing pancreatitis patients with or without organ failure**

|  | **All patients(n=432)** | **Organ**  **failure=302(69.9%)** | **No**  **Organ failure=130(30.1%)** | **P**  **values** |
| --- | --- | --- | --- | --- |
| **Pancreatic necrosis** |  |  |  | 0.000† |
| ANC | 338(78.2%) | 218(72.2%) | 120(92.3%) |  |
| ANC infection | 71(16.4%) | 67(22.2%) | 4(3.1%) |  |
| WON | 10(2.3%) | 6(2.0%) | 4(3.1%) |  |
| WON infection | 13(3.1%) | 11(3.6%) | 2(1.5%) |  |

**Abbreviations:** Walled-off necrosis=WON. Acute necrosis collection=ANC.

**Supplementary Table 3.** **Characteristics of Acute necrotizing pancreatitis patients died or not**

|  | **All patients(n=432)** | **death=44(10.2%)** | **No death=388(89.8%)** | **P**  **values** |
| --- | --- | --- | --- | --- |
| **Pancreatic necrosis** |  |  |  | 0.000† |
| ANC | 338(78.2%） | 18(40.9%） | 320(82.5%） |  |
| ANC infection | 71(16.4%） | 20(45.5%） | 51(13.1%） |  |
| WON | 10(2.3%） | 0 | 10(2.6%） |  |
| WON infection | 13(3.1%） | 6(13.6%） | 7(1.8%） |  |
| **Respiratory failure** | 298（69.0%） | 44（100.0%） | 254（65.5%） | 0.000† |
| **renal failure** | 87（20.1%） | 34（77.3%） | 53（13.7%） | 0.000‡ |
| **Circulatory failure** | 8（1.9%） | 6（13.6%） | 2（0.5%） | 0.000† |

**Abbreviations:** Walled-off necrosis=WON. Acute necrosis collection=ANC.

**Supplementary Table 4.** **Univariate and multivariate analyses of risk factors for organ failure in acute necrotizing pancreatitis**

| **Variable** | **Univariate analyses** | | **Multivariate analyses** | |
| --- | --- | --- | --- | --- |
|  | **OR (95% CI)** | **P** | **OR (95% CI)** | **P** |
| **Pancreatic necrosis** |  |  |  |  |
| ANC infection | 0.09(0.02-0.45) | 0.003 | 0.19(0.03-1.44) | 0.108 |
| ANC | 0.83(0.23-2.98) | 0.770 | 0.57(0.11-2.96) | 0.503 |
| WON infection | 0.27(0.04-1.95) | 0.196 | 0.67(0.05-9.80) | 0.770 |
| WON | Ref (1.00) |  | Ref (1.00) |  |

**Abbreviations:** Walled-off necrosis=WON. Acute necrosis collection=ANC.

**Supplementary Table 5. Univariate and multivariate analyses of risk factors for death in acute necrotizing pancreatitis**

| **Variable** | **Univariate analyses** | | **Multivariate analyses** | |
| --- | --- | --- | --- | --- |
|  | **OR (95% CI)** | **P** | **OR (95% CI)** | **P** |
| **Pancreatic necrosis** |  |  |  |  |
| ANC infection | 0.00(0.00- ) | 0.999 |  |  |
| ANC | 0.00(0.00- ) | 0.999 |  |  |
| WON infection | 0.00(0.00- ) | 0.999 |  |  |
| WON | Ref (1.00) |  |  |  |
| **Respiratory failure** | 0.00(0.00- ) | 0.996 |  |  |
| **renal failure** | 0.05(0.02-0.10) | 0.000 | 0.50(0.01-17.9) | 0.703 |
| **Circulatory failure** | 0.03(0.01-0.17) | 0.000 | 0.08(0.00-1.59) | 0.098 |

**Abbreviations:** Walled-off necrosis=WON. Acute necrosis collection=ANC.
